# Milling Performance of CFRP Composite and Atomised Vegetable Oil as a Function of Fiber Orientation

**DOI:** 10.3390/ma14082062

**Published:** 2021-04-20

**Authors:** Tarek-Shaban-Mohamed Elgnemi, Martin Byung-Guk Jun, Victor Songmene, Agnes Marie Samuel

**Affiliations:** 1 Department of Mechanical Engineering, École de Technologie Supérieure (ÉTS), Montreal, QC H3C 1K3, Canada; tarek-shaban-mohamed.elgnemi.1@ens.etsmtl.ca (T.-S.-M.E.); agnesmsamuel@gmail.com (A.M.S.); 2 Department of Mechanical Engineering, Purdue University, West Lafayette, IN 47907, USA; mbgjun@purdue.edu

**Keywords:** CFRP, dry/vegetable oil, cutting force, delamination, tool edge rounding

## Abstract

Carbon fiber reinforced polymers (CFRPs) have found diverse applications in the automotive, space engineering, sporting goods, medical and military sectors. CFRP parts require limited machining such as detouring, milling and drilling to produce the shapes used, or for assembly purposes. Problems encountered while machining CFRP include poor tool performance, dust emission, poor part edge quality and delamination. The use of oil-based metalworking fluid could help improve the machining performance for this composite, but the resulting humidity would deteriorate the structural integrity of the parts. In this work the performance of an oil-in-water emulsion, obtained using ultrasonic atomization but no surfactant, is examined during the milling of CFRP in terms of fiber orientation and milling feed rate. The performance of wet milling is compared with that of a dry milling process. The tool displacement-fiber orientation angles (TFOA) tested are 0°, 30°, 45°, 60°, and 90°. The output responses analyzed were cutting force, delamination, and tool wear. Using atomized vegetable oil helps in significantly reducing the cutting force, tool wear, and fiber delamination as compared to the dry milling condition. The machining performance was also strongly influenced by fiber orientation. The interactions between the fiber orientation, the machining parameters and the tested vegetable oil-based fluid could help in selecting appropriate cutting parameters and thus improve the machined part quality and productivity.

## 1. Introduction

Carbon fiber reinforced polymers (CFRPs) find many industrial applications (automotive, aerospace, sporting goods, medical and military) because of their attractive specific strength, specific modulus, damage tolerance characteristics [1,2], and resistance to fatigue and corrosion [3,4]. Despite their promising outlook, the machinability of CFRPs has often been poor because of excessive tool wear [5,6], machining-induced delamination [7,8], fiber fragmentation, pull-out, and spalling [9,10]. In particular, abrasive carbon fibers rapidly wear out the tool, changing its geometry and damaging the subsurface of the work piece [11]. Alternative machining strategies must be found to shape CFRP parts with acceptable tool life, good part quality/accuracy and less surface and subsurface damages. Considerable improvement has been accomplished in terms of comprehension of machinability during milling of isotropic materials, such as metals [12,13]. In spite of this research, outcomes cannot be immediately applied to the milling of CFRP laminates due to the current inhomogeneity and anisotropy of these materials [14]. Unlike minerals that are outwardly homogeneous, where machining is a matter of plastic deformation and shear, the machining of CFRPs is related to plowing, cutting, and cracking [15,16].

Structural CFRP parts are shaped using a milling process and then drilled in order to assemble them (riveting, bolting) with other components [17,18]. Most researchers recognize the difficulties in applying knowledge obtained during machining of metals or other types of material to CFRPs because of inhomogeneity, anisotropy [12,13], and damages occurring during cutting [14]. In fact, during machining of CFRPs, plowing, cutting, and cracking occur [16,19], whereas the machining of metals takes place by shearing and plastic deformation [20]. The machining of CFRPs is also influenced by the machining parameters and conditions which affect the cutting force/temperature and, therefore, the tool wear and part quality [21,22]. To overcome these difficulties, several studies have investigated the machining of CFRPs in order to propose optimal machining parameters and conditions such as cutting speed [23,24], feed rate [25,26], and depth of cut [27,28]. One of the most interesting results from these studies was that machinability and tool wear processes strongly depend on fiber-orientation corresponding to the cutting direction. It has also been recognized that the subsurface damage and high surface roughness associated with machining often results in poor surface quality that is unacceptable for design [29,30]. The effects of material and machining conditions on tool wear have been investigated in the drilling of CFRP [31,32]. The results show an improvement in the drilled hole quality. These works demonstrate, among other things, the influence of cutting direction relative to fiber orientation on the machinability of CFRPs, particularly on tool wear and part quality. Part subsurface damage and high surface roughness are unacceptable in the use of these composites [25].

Cutting fluids are usually used for improving tool life, surface finish and lowering cutting forces when machining metallic materials. They also flush chips away from the cutting zone and reduce dust generated in the working environment, thus improving occupational safety. In the case of CFRPs, this is inadvisable because of the negative impact on the structural integrity of the composite [20].

An innovative method (fully wet or semi-wet) that can cool down the tool [33] or lubricate the cutting process without being absorbed by the workpiece material is feasible for the machining of CFRPs [34,35]. This is the case for cryogenic machining, which was found to improve tool life and part surface integrity during drilling of CFRPs [36,37]. In recent years, some authors [38,39] have studied tool wear in the milling and edge-trimming of CFRPs using chilled air and dry cutting conditions. They observed that using chilled air improved the tool life of uncoated carbide cutting tools compared to dry machining, provided the feed rate was selected with care, this being an influential process parameter.

Moreover, vegetable-based oils have also been proposed as a viable alternative [40,41] as they are environmentally friendly and show highly suitable lubricity and other characteristics. These vegetable oils include canola, soybean, and rapeseed oil. Research on the development and formulation of vegetable oil-based metal working fluids (MWFs) can be found in [41], while their performance is reported in [42,43]. These vegetable-based oils appear definitively as a good alternative to mineral-based cutting oils [44,45]. Ultrasonic vibration can be used to emulsify vegetable oil in water [46,47]. However, the feasibility of using such fluid as an MWF for machining has not been investigated, with the exception of the authors’ previous works [48,49]. The performance of such a cutting fluid on CFRPs was studied in [48] and it was reported that this fluid effectively reduced tool wear, cutting forces, surface roughness, burr occurrence, and dust and airborne concentrations in the machining environment. However, the study did not address the effect of fiber orientation.

Therefore, this work is mainly motivated by two objectives: (i) the need to study the performance of ultrasonic atomized vegetable oil in water as a sustainable alternative to conventional cutting oils and coolants; and (ii) to study the impact of fiber orientation on the tool degradation mode (edge rounding); the quality of the machined surface, delamination percentage and fiber length are also considered in regard to the latter.

The article is organized as follow: Section 2 describes the experimental setup, workpiece, cutting tool and the design of experiments. The results (cutting forces, friction angle, tool wear, and delamination) are presented and discussed in Section 3. The concluding remarks are given in Section 4.

## 2. Methodology

### 2.1. Machining Process Setup

**Figure 1:**
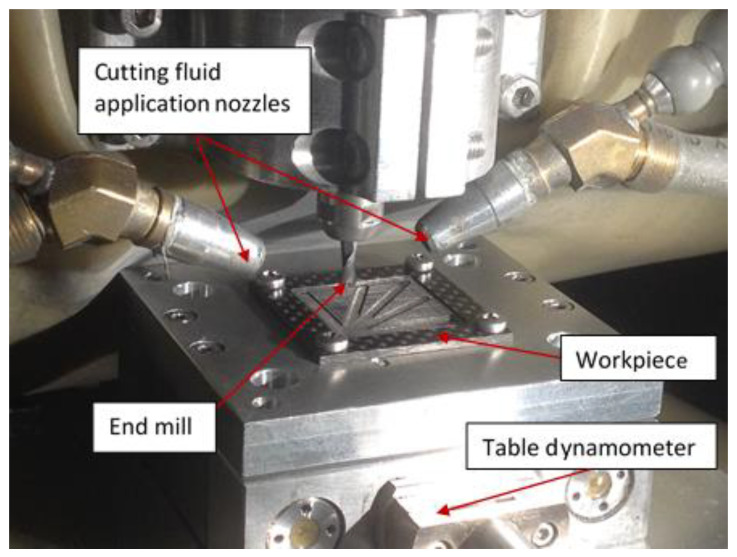
Experimental set-up consisting of a workpiece mounted on table dynamometer, end mill cutting tool and cutting fluid application nozzles.

The system of atomization-based cutting fluid spray is similar to the one given by Elgnemi et al. in [48]. The atomized canola vegetable oil and water is directly applied to the cutting zone to lubricate the chip and tool interface. The average droplet size of the canola oil is around 2.1 µm [49]. More details are available in [50,51]. Slot milling tests are carried out under cutting conditions as shown in Figure 1. The machine-tool used in this work was ALIO vertical milling machine (supplied by Alio Industries) which has a spindle speed range of 10,000 to 80,000 rpm. The workpiece was clamped on the table dynamometer fixed on the machining center. Cutting forces were measured using a Kistler MiniDyn 9256C1 dynamometer as shown in Figure 1. A data acquisition (DAQ) board from National Instruments (NI-P/N-USB-6251 BNC) was used to acquire the measured forces from the dynamometer. The CutPro software program (developed by Prof Y. Altintas’ team, University of British Colombia, Vancouver, Canada) was used to receive data from the DAQ board during the machining operation. A sampling rate of 100 kHz was used to process the measured force data. For comparison purposes, the experiments were carried out in both dry conditions and wet conditions using canola vegetable oil.

In the latter case, distilled water was added into the atomizer to dilute the fluid at 5 vol.%.

### 2.2. Workpiece Materials and Cutting Tool

Multi-layer CFRP sheets 1.56 mm thick were used as workpiece material in this study. Each cut was done at a unidirectional fibers orientation with a depth of cut of 0.3 mm. Each composite sheet consisted of four unidirectional tapes (54 vol.% fibers per tape) laid up in 0° and 90° orientations. The combined Young’s modulus of 104 fibers (in the sheets) was 225 GPa (65 GPa per tape). The workpiece materials were cut into small sheets of 38 mm length × 38 mm width. The tests were performed on unidirectional laminates and the cutting directions were set so that the fiber orientation angle with respect to the cutting direction was 0°, 30°, 45°, 60° and 90.

The tool geometry information for the uncoated tungsten carbide (WC-Co) 2-flutes end-mills (MSC Industrial Supply, Elkhart, IL, USA) used in the experiment are summarized in Table 1.

### 2.3. Measurement of Cutting Forces and Friction Angle

The forces *Fc*, *Ft* and *R* are the main cutting force, thrust force and resultant cutting force, respectively. They are calculated from the measured normal force *Fx* and the feed force *Fy* in the *x* and *y* directions. The magnitude of *Fz* was negligible compared to *Fx* and *Fy*. The relationships between these forces are: [52]

(1) Fc=−Fx sin ∅+Fy cos ∅

(2) Ft=Fx cos ∅+Fy sin ∅ 

(3) $$R=\sqrt{Fc^{2}+Ft^{2}}$$

Since the un-deformed thickness of the chip t is almost the same as the depth of cut used, (given the brittle nature of the CFRP [53,54]), the shear angle ∅ can be estimated through the tool rake angle (*α*) by:

(4) $$\emptyset \approx \;tan^{-1}\;\left(\frac{cos\alpha }{1-sin\alpha }\;\right)$$

and the friction angle (*β*) by [52]:

(5) $$tan\;\left(\beta \right)=\frac{Ft+Fc\;tan\alpha }{Fc-Ft\;tan\alpha }$$

### 2.4. Edge Rounding Measurement

**Figure 2:**
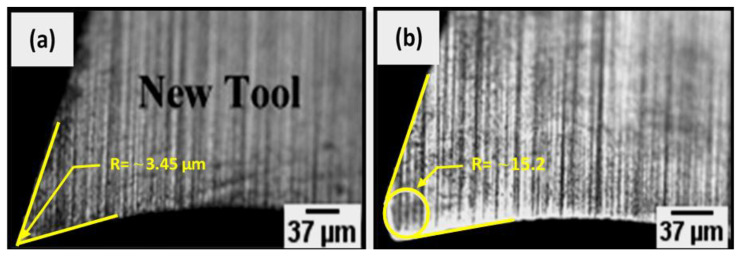
Procedure for measuring the tool nose radius. (**a**) New tool, (**b**) worn tool for a cutting distance of 66 mm with 0° TFOA at a feed rate of 3 µm/tooth and 20,000 rpm, in the dry condition.

The effective cutting-edge radius affects the machining performance (forces, power requirements) and the part surface finish. In this study, changes in cutting edge evolution were taken into consideration. A 3D optical microscope (Olympus BXFM) was employed for monitoring the change in the tool point or nose radius. Microscope images of a sharp cutting-edge are shown in Figure 2a and microscope image of the same cutting edge after milling three slots (total cutting distance of 66 mm) is shown in Figure 2b. The tools were cleaned in acetone in an ultrasonic bath at 60 °C for 30 min (frequency: 1000 kHz, power: 60 W) [55,56], and [57]. The radius was estimated using the best-fit radius (highlighted circle) between the flank and the rake face of the worn tool. The tangential intersection with the flank face and the tangential intersection with rake face represent a series of radiuses measured from the worn cutting-edge profile between flank and rake face, as shown in Figure 2b. The radius of the circle that is confined between the two lines is considered as the resulting tool wear.

### 2.5. Delamination Measurement

**Figure 3:**
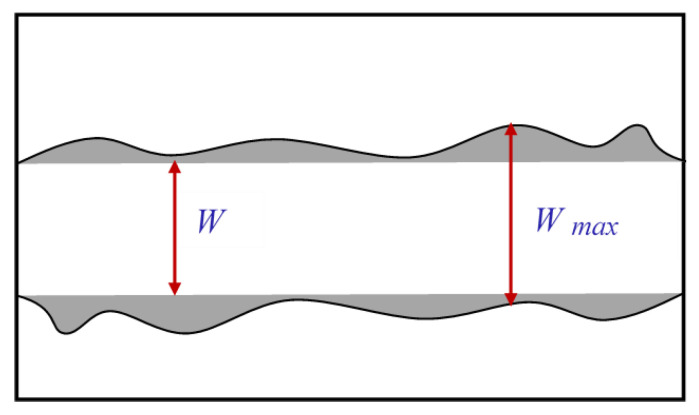
Schematic representation of CFRP damage measurement technique.

Delamination is a common occurrence when milling CFRPs. This machining-induced damage can be estimated using the delamination percentage *Dp*, measured using the maximum damage width (*W_max_*) and the programmed slot width (*W*), corresponding to endmill diameter in Equation (6) [58]. *W_max_* was estimated using optical microscope observations. Figure 3 shows a schematic representation of these terms.

(6) $$Dp=100\frac{Wmax-W}{W}\;$$

### 2.6. Chip Formation Characterization Processes

**Figure 4:**
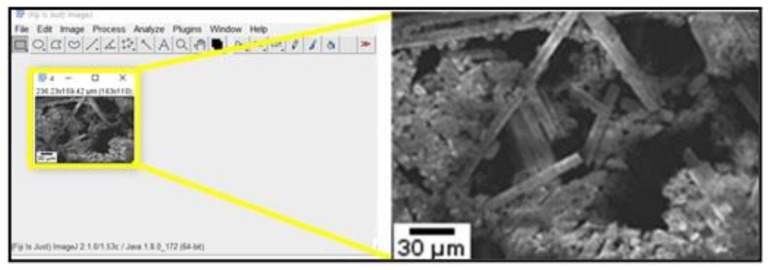
Fiber length measurement technique.

After machining, chips were collected for each of the cutting directions (0°, 30°, 45°, 60°, and 90°) relative to fiber orientations (TFOA) of CFRP. These chips were then measured and imaged employing a Hitachi S-4800 FESEM. Figure 4 shows the technique for measuring the fiber length as well as examples of chip morphologies observed. In this image, we can distinguish fibers separated from the matrix. Broken short fibers may have been wiped out during the cleaning procedure. The lengths of exposed long fibers were estimated using Image J software. 

### 2.7. Experimental Design

**Figure 5:**
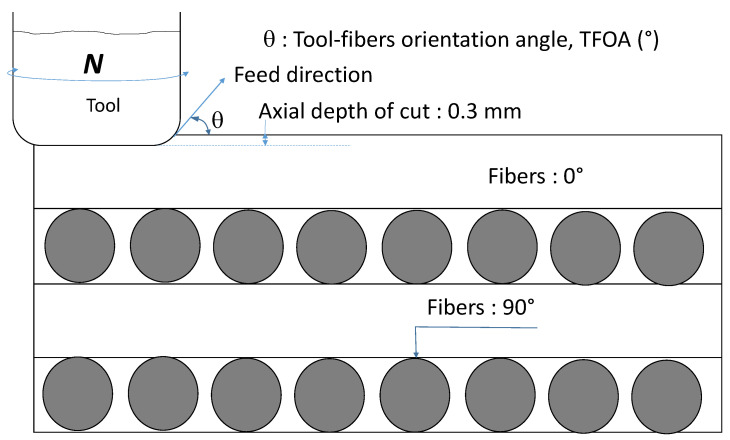
Schematic representation of tool displacement-fibers orientation angle.

A factorial design approach was implemented to look into the milling parameters and cooling method effects on the machining of CFRP. The tested factors included: (a) the fiber orientation angle with respect to the cutting direction as shown in Figure 5; (b) the feed rate; and (c) the cooling method. Five fiber orientation angles (TFOA: 0°, 30°, 45°, 60°, and 90°), two feed rates and two cooling methods (dry versus atomized vegetable oil) were selected. In all, twenty experiments were necessary for the full factorial design. Each test was repeated three times. A new tool was used for each test. Table 2 summarizes the controlled factors, their level and the output responses analyzed. The others milling parameters were set as follows:

- Spindle speed: 20,000 rpm
- Tool immersion: 100%
- Axial depth of cut: 0.3 mm
- Length of each slot: 22 mm

The depth of cut was chosen so as to be able to make three passes on the same fiber orientation.

## 3. Results and Discussion

### 3.1. Cutting Forces

**Figure 6:**
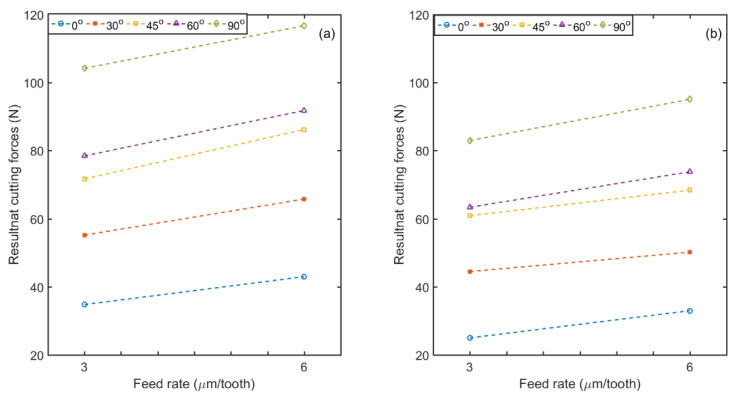
Influence of feed rate and fiber orientation on resultant cutting forces.in the (**a**) dry, and (**b**) ACF vegetable oil conditions (at 20,000 rpm speed).

**Figure 7:**
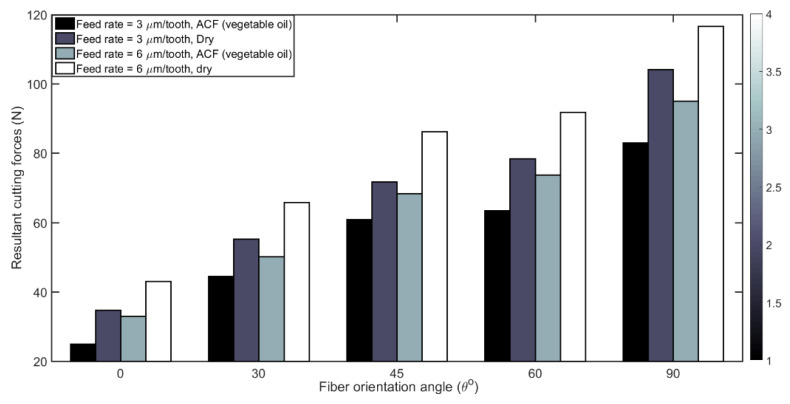
Resultant cutting forces for all fiber orientations using 3 and 6 µm/tooth feed rates and different cutting conditions. (Speed: 20,000 rpm).

The influence of feed rate and fiber orientation on the resultant cutting force (*R*) obtained when using dry and ACF (atomized cutting fluid i.e., vegetable oil) milling conditions are shown in Figure 6. For all tested parameters, a slight increase in *R* was observed with dry milling of uni-directional or UD-CFRP laminates compared to atomization-based spray machining regardless of TFOA and feed rate. This can be attributed to the lubricating effect due to the ACF resulting in reduced friction at the tool/chip and tool/work piece interfaces when atomized cutting fluid is used. Moreover, in Figure 7 the highest magnitude of *R* is noted at 90° fiber orientation, whereas the *R* value is lowest for 0° orientation, namely when the cutting direction and fibers are parallel. In such a setting, the main mechanism for chip formation is delamination, and therefore the interaction of fibers with the cutting edge is limited. In the opposite case, when the fibers are perpendicular to the cutting direction (i.e., at 90° TFOA), the tool must cut the fibers that have a higher Young’s modulus than the matrix. For the 45° fiber orientation, the fibers undergo both pulling and shearing [57]. It appears in Figure 6 that the resultant force increases proportionally to the feed rate, as usually expected when machining metals, polymers and composites since the chip thickness and cross section, and therefore the metal removal rates, are increased at a high feed rate [59]. The results presented in Figure 6 and Figure 7 demonstrate that the machining of CFRP is improved with the use of the atomization-based vegetable oil spray as the lubricant.

### 3.2. Friction Angle

**Figure 8:**
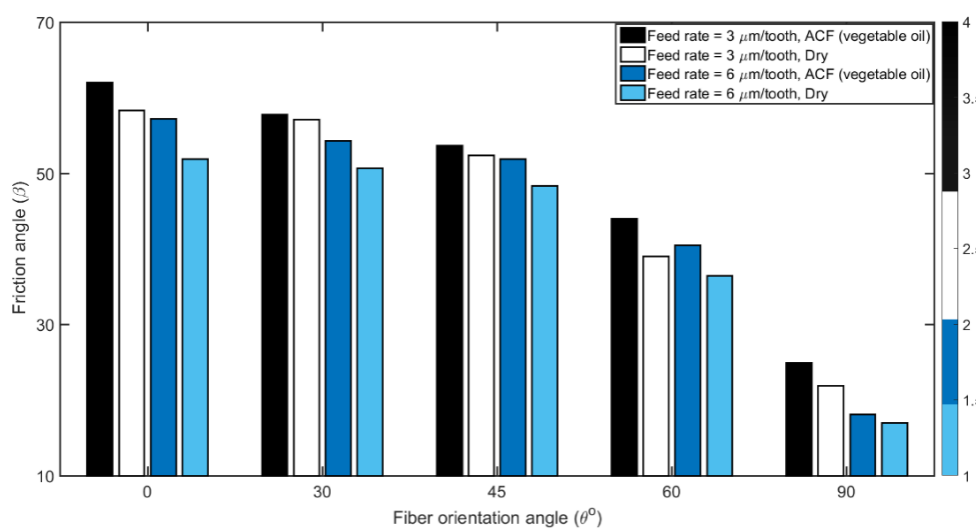
Comparison of measured friction angle as a function of fiber orientation at feed rates with 3 and 6 µm/tooth and different cutting conditions (at 20,000 rpm speed).

The bar diagram in Figure 8 illustrates the friction angle by feed rate and fiber orientation. The friction angle β is plotted according to Equation (5). Regardless of the dry or lubricated conditions, it appears that the friction angle decreases as the cutting direction changes from 0° to 90° orientation. The reduction in β is limited from 0° to 45° and high when cutting in fiber orientation between 60° to 90°. This can be explained by the fact that, for high fiber angles (more than 45°), the double fiber removal actions of pulling and shearing lead to reduction in chip-tool contact length and thus reduce the friction. The rupture of fibers strongly depends also on the TFOA as well as the effective rake angle α, Equation (5) [60]. Figure 8 also shows that β decreases proportionally with an increase in the feed rate (i.e., chip thickness) for each unidirectional CFRP laminate when the cutting conditions are the same. This observation may be ascribed to the reduction in the un-deformed chip thickness which depends on the amount of feed per tooth; as a result the friction angle β at the tool face will decrease as the feed per tooth is increased [61]. The other differences in friction angles observed, especially as a function of TFOA, could be attributed to the chip formation process [53]. Finally, the use of ACF lubricant will also reduce the friction angle. Moreover, referring to Equation (5), the theoretical friction angle would decrease with a higher cutting force. The parameters affecting the machining force, as described in the previous sub-section, further corroborates the correlation between the increase in cutting force and the decrease in friction angle.

### 3.3. Tool Wear-Edge Rounding

**Figure 9:**
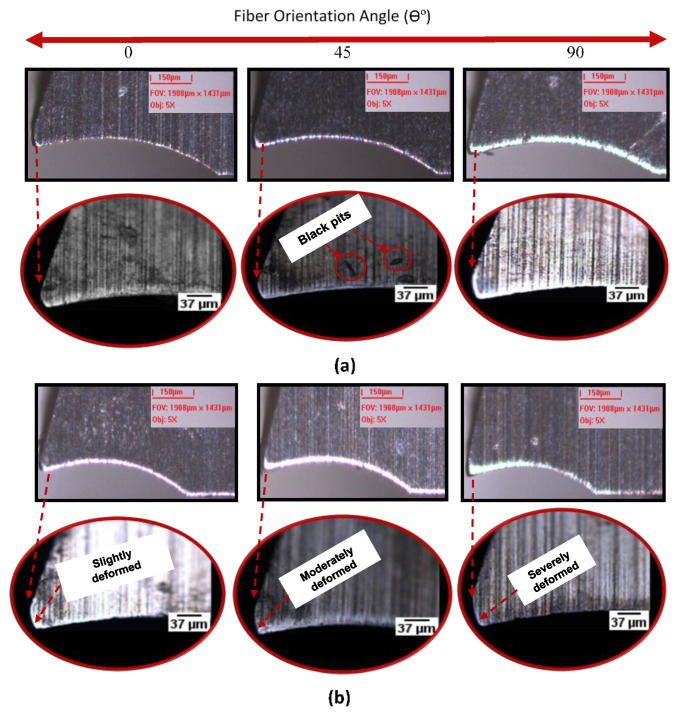
Progression of wear after milling 66 mm at 0°, 45°, and 90° fiber oriented at feed rate 3 µm/tooth, and 20,000 rpm using (**a**) atomization-based spray and (**b**) dry milling.

**Figure 10:**
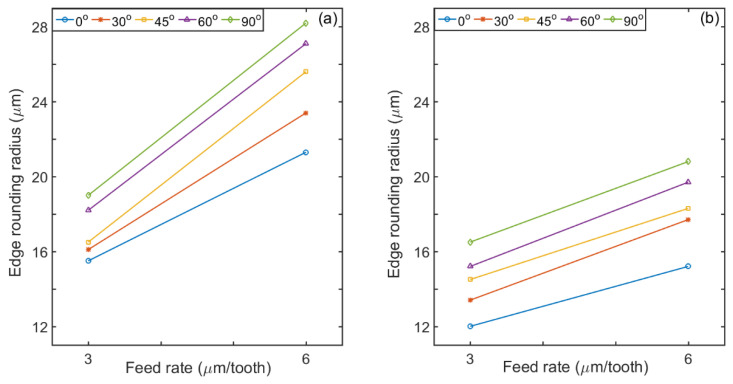
Comparison of measured edge rounding radius progress for all fiber orientations at feed rates 3 and 6 µm/tooth and speed of 20,000 rpm for (**a**) dry and (**b**) ACF vegetable oil conditions.

The dominant tool wear mode when machining CFRP has already been identified as the blunting of the cutting edge [62,63], also known as edge rounding [64,65]. Figure 9 shows comparative images of the tool after milling three slots (total travel distance of 66 mm) under different lubricating conditions and for tested fiber orientation angles. The higher the fiber orientation, the higher the edge rounding observed after milling. Figure 10 presents a comparison of edge rounding in terms of fiber orientation, milling condition and feed rate. It appears from the trends of Figure 9 that, irrespective of fiber orientation angles and feed rate values, the cutting edges develop a large edge radius caused by the abrasion of the tool flank and rake faces by the broken carbon fibers. Figure 10 displays how the measured tool edge rounding radius varies with the feed per tooth and the use (or not) of cutting fluid. From this figure, it is noted that tool wear is much greater when no lubricant was used, due to the increase in friction.

In general, the increase in edge rounding radius was high for cutting fibers at 90° (i.e., perpendicular to the cutting direction) and least for fibers cut at 0°. This may be attributed to shear fracture that occurs during CFRP chip formation when cutting with 90° as reported by Maegawa et al. [66]. This result also explains the highest cutting force observed at 90° fiber orientation (Section 3.1) During machining at 45°, the cutting force was not as high as for 90° (Figure 6 and Figure 7). These results are in keeping with those reported by Wang et al. [25].

The low edge rounding radius observed at 45° and 0° may be accounted for in terms of the chip formation. When milling at 45°, the abrasive action of the broken fibers is high. These fibers are broken because of the compression-induced shear perpendicular to the fiber direction [59]. When machining at 0°, fiber delamination occurs because of micro-buckling and only limited fibers can fracture [67].

It is also observed in Figure 10 that the tool deterioration (edge rounding) was reduced during milling for all cutting directions using atomized vegetable oil conditions. It can be observed too, for all lubrication conditions tested, that the tool wear increases when the feed rate and thus the load on the tool is increased.

### 3.4. Delamination

**Figure 11:**
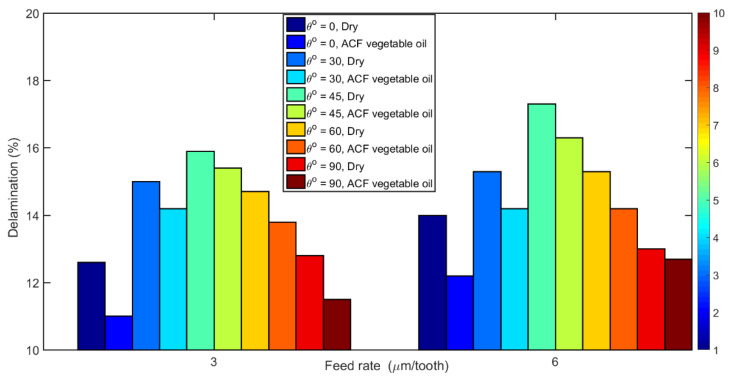
Variation in delamination percentage with fiber orientation angle at feed rates 3 and 6 µm/tooth and different cutting fluid conditions (at 20,000 rpm speed).

Figure 11 displays the percentage of delamination measured (Equation (6)) as a function of TFOA and feed rate on samples machined under dry and wet conditions. It can be seen that, when milling using the atomized coolant, the percentage of damage on the workpiece is greatly reduced for all the tested conditions. The resulting reduction of temperature in the cutting zone can explain this behavior since it would reduce the thermal degradation of the polymer matrix. This result is in good agreement with the observations of Kumar et al. [59] who found that low temperatures tend to maintain or increase the properties of the CFRP (tensile strength, shear modulus and stiffness) which, in consequence, would produce greater resistance to delamination.

The maximum damage occurred when cutting dry at 45° fiber orientation using a high feed rate (Figure 11). The dominant chip formation mechanisms, i.e., fiber bending and shearing, for this configuration can explain this maximum delamination. Also, the more the feed rate is high, the higher the delamination that takes place, which can be attributed to the increase in cutting forces and thus on shear stress when the feed rate is increased.

### 3.5. Chip Formation

**Figure 12:**
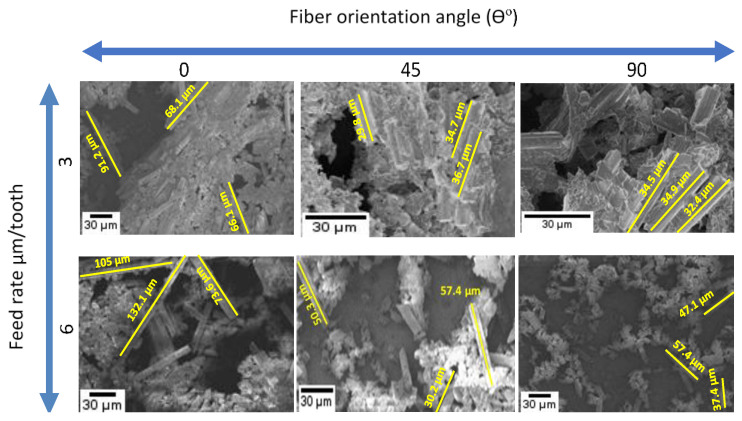
SEM images used for fiber length measurements for fiber orientations θ° = 0, 45, and 90 at feed rates 3 and 6 µm/tooth and dry milling condition (at 20,000 rpm speed).

In general, the chip formation observed during milling of CRPFs is dominated by delamination and breakage. The reinforcing fibers break into dust particles of different shapes and this tendency varies depending on the direction of cutting relative to the fiber orientation. Figure 12 shows SEM images of fibers and chips obtained, in relation to the feed rate and TFOA. Measurements of broken fibers were taken from these images to ascertain their lengths in order to correlate them with the machining conditions (see Figure 12).

**Figure 13:**
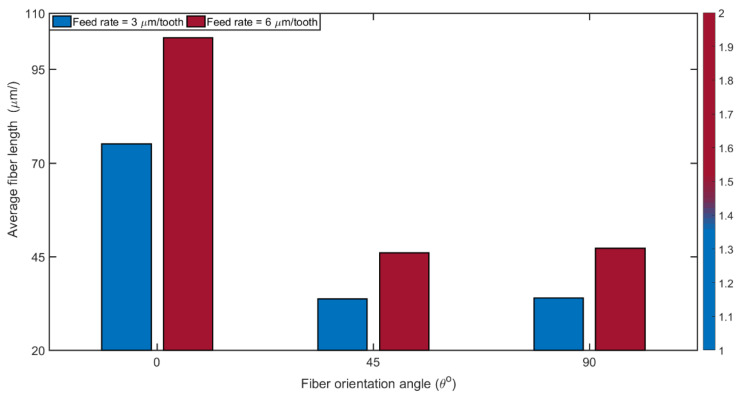
Average fiber length measurements of 0°, 45°, and 90° fiber orientations at feed rates 3 and 6 µm/tooth and dry milling condition (at 20,000 rpm speed).

It is clearly noted in Figure 12 that the chips are broken into fiber segments of irregular lengths. Longer broken fibers were obtained at 0° TFOA while much shorter ones were generated at 45° and 90° TFOAs. The chip formation modes at 0° TFOA (i.e., delamination [16,57]) and at 45° and 90° TFOAs (i.e., compression-induced shear fracture perpendicular to fibers and inter-laminar shear fracture along the fiber/matrix interface [16,68] can explain the difference found in the broken fiber lengths. These broken fibers abrade the tool, which explains the high edge radius roundness observed when milling at 0° orientation (see Figure 10). It was also observed from Figure 13 that fiber length increases proportionally to the feed rate (i.e., chip thickness) for all fiber orientations. For instance, the average fiber length increased, from 75.1 μm at a feed rate of 3 μm/tooth with 0° orientation to 103.5 μm at feed rate 6 μm/tooth with 0° orientation.

### 3.6. Impact of Fiber Orientation on CFRP Surface Finish

When CFRPs are subjected to milling, the formed surface is irregular because of the pulling and separation of the fibers from the matrix [69]. The irregular surface is also the result of variation in cutting forces when the tool cuts the fibers and then the matrix. It has been reported previously that surface damage when milling CFRP occurs in the transition zone [70,71,72].

**Figure 14:**
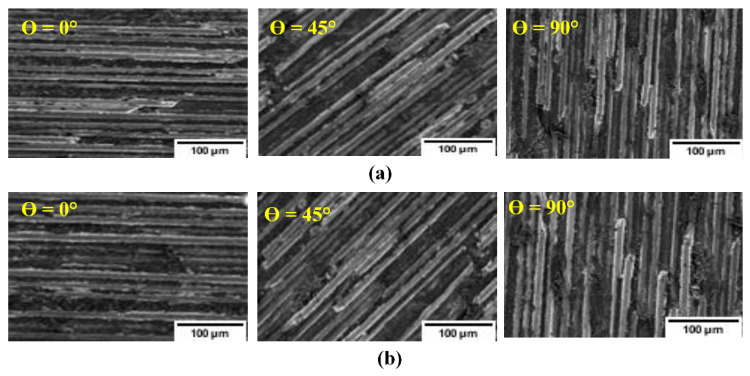
SEM images of a machined surface obtained after milling 0°, 45°, and 90° TFOA (feed rate µm/tooth and speed of 20,000 rpm): (**a**) ACF vegetable oil conditions and (**b**) dry.

Figure 14 illustrates the surface damage of unidirectional CFRP laminate at 0°, 45°, and 90° TFOAs. The TFOA appears to be the dominant factor for the surface generated (Figure 14) as the surfaces for the 45° and 90° orientations are more severely damaged than that for 0° orientation. In fact, at 0° TFOA, the cutting orientation is parallel to the fibers’ orientation and the major chip formation mechanism is delamination, so there is minimum interaction between fibers and cutting edge. For the 90° TFOA, the cutting orientation is orthogonal to the fibers, causing the greater interaction between fibers and the cutting edge. For 45°, the fibers are both dragged and sheared. This behavior is well known in the literature [73,74]. In the case of fibers oriented at 45° and 90°, the fibers were not completely removed because of the large bending deformation taking place. It can be thus concluded that during machining of CFRP, surface damage is related to the chip formation mechanism where bending and shearing of fibers dominate the mechanism [60].

Moreover, the TFOA seriously affects the tool cutting-edge wear, as a result of surface damage occurring within the workpiece during machining. The analysis of tool edge radius was described in Section 3.3. The results confirm the high effect of fiber orientations on tool wear. At 0° fiber orientation, the surface integrity is much better than at 45° or 90° TFOA. In fact, at 0°, crushing and then fracture of fibers is dominant and therefore fibers crack or fracture at the cut surface. Instead, for other TFOAs (between 45° and 90°), fracture occurs ahead of the tool, leading to further damage of the surface as a result of the uncontrolled action of the broken fibers.

Nevertheless, it should be noted that employing ACF vegetable oil did not show a pronounced effect on the machined surface when compared to dry milling. The use of ACF vegetable oil, however, as a spray in the cutting zone, is a viable lubricating method for CFRP since the spray will evaporate and will not be absorbed by the workpiece. Thus, the structural integrity of the CFRP will not be damaged because of moisture from the lubricant. This is in agreement with our earlier findings [48,49].

## 4. Conclusions

The milling performance of CFRPs when using atomized vegetable oil as a lubricant was investigated in this research work as a function of fiber orientations and cutting feed rate. The process performance indicators studied were the cutting force, the tool wear, delamination, and surface texture. The results obtained from this study led to the following conclusions:

(1) When machining CFRPs, the cutting direction, in relation to the fiber orientation, strongly influences chip formation, on which depends resistance to cutting, cutting force, fiber breakage, tool wear, the quality of the machined surface and the delamination. These indicators depend on the machining parameters used and on the cutting conditions.
(2) The delamination percentage when cutting at TFOAs of 0°, 30° 45°, 60°, and 90° were improved by 65%, 91%, 54%, 66%, and 75%, respectively, under ACF (vegetable oil) conditions at 3µm/tooth. Machining with 45° TFOA produces maximum damage at high feed rate.
(3) As expected, increase in feed rate increased the cutting force because of high chip load, especially in cutting directions where the fiber strength is high and with dry machining. Using a lubricant decreased the friction between the chip and the rake face of the tool, reduced the abrasive effect of broken fibers, and thus lowered cutting force and tool wear.
(4) The magnitude of resultant cutting force was found to be greater in the dry condition relative to the ACF condition by about 23%, 31%, 26%, 25%, and 23%, respectively, for the samples in the 0°, 30, 45°, 60°, and 90° TFOA at 6 μm/tooth of feed rate. However, the use of atomized vegetable oil mixed with water improved the process performance compared to dry machining in that it reduced the cutting force, the delamination percentage and the tool edge rounding.
(5) The tool damage was examined and measured using a 3D optical microscope (Olympus BXFM). 90° TFOA produced a large amount of tool edge rounding. Contrastingly, 0° TFOA has the least amount of edge rounding. However, the lowest values of edge radius evolution as a measure of the tool wear were 12 µm and 16.5 µm with TFOA from 0° to 90° at feed rate 3 µm/tooth under ACF (vegetable oil) conditions.
(6) The findings of this study further support the use of the vegetable oil-in-water emulsion obtained through ultrasonic atomization as an effective and environmentally friendly lubricant for improving the machining of CFRPs.

## Figures and Tables

**Table 1:** Description of the geometry of end mill used.

Cutter Diameter	Flute Length	Shank Diameter	Overall Length	Rake Angle	Helix Angle
0.125 in	0.250 in	1/8 in	1.50 in	7°	30°
3.175 mm	6.35 mm	3.175 mm	38.1 mm		

**Table 2:** Details of milling experiments with and without atomization-based cutting fluid (ACF).

Ident.	Control Variables	Levels	Response Variables
A B C	Fiber orientation (Ɵ°) Feed rate (µm/tooth) Cutting fluids	0, 30, 45, 60, 90 3, 6 Dry-ACF (vegetable oil)	1. Cutting forces 2. Tool wear 3. Delamination 4. Surface damage 5. Chip formation

## Data Availability

Not applicable.

## Nomenclature

CFRP	carbon fiber reinforced polymer	rpm	revolution per minute
ACF	atomization cutting fluid	∅	shear angle (°)
TFOA	Tool displacement direction-fiber orientation angle: θ (°)	*α*	rake angle (°)
*Fx*	normal force in the x direction	*β*	friction angle (°)
*Fy*	feed force in the y direction	N	newton
*Fz*	force in the z direction	µm	micrometer
*Fc*	cutting force	*Dp*	delamination percentage (%)
*Ft*	thrust force	*W_max_*	maximum damage width (mm)
*R*	resultant cutting force	*W*	original slot width (mm)
